# Hashimoto’s Thyroiditis Effects on Papillary Thyroid Carcinoma Outcomes: A Systematic Review

**DOI:** 10.7759/cureus.28054

**Published:** 2022-08-16

**Authors:** Darin Osborne, Rabia Choudhary, Abhishek Vyas, Prathima Kampa, Lawahiz F Abbas, Himaja Dutt Chigurupati, Michael Alfonso

**Affiliations:** 1 Internal Medicine, California Institute of Behavioral Neurosciences & Psychology, Fairfield, USA; 2 Family Medicine, California Institute of Behavioral Neurosciences & Psychology, Fairfield, USA; 3 Research, California Institute of Behavioral Neurosciences & Psychology, Fairfield, USA; 4 Medicine, California Institute of Behavioral Neurosciences & Psychology, Fairfield, USA

**Keywords:** autoimmune disease, thyroid cancer, chronic lymphocytic thyroiditis, papillary thyroid carcinoma, autoimmune thyroiditis, papillary carcinoma of thyroid, hashimoto’s thyroiditis

## Abstract

Papillary thyroid carcinoma (PTC) and Hashimoto’s thyroiditis (HT), also known as chronic lymphocytic thyroiditis, are both common thyroid diseases that are increasing in prevalence. PTC is well-differentiated cancer that generally has an excellent prognosis. HT is an autoimmune disease that often leads to hypothyroidism. A significant proportion of PTC patients also have HT. This systematic review will analyze the effect of HT on the characteristics and outcomes of PTC.

Several databases were systematically searched using relevant medical subject headings (MeSH) keywords and phrases examining the connection between PTC and HT and the effect of their coexistence. Inclusion and exclusion criteria were applied, followed by quality appraisal. After that filtration process, 23 articles were selected with a total of 41,646 patients.

Out of 22 studies commenting on tumor size, 12 studies demonstrated it to be smaller in HT patients, while 10 studies observed no effect. Eleven studies examined PTC angioinvasion, most of which found no difference in HT and non-HT patients. However, two studies found angioinvasion to be reduced in PTC patients. As for capsular infiltration, out of the five studies commenting on it, two found decreased occurrence, one found increased occurrence, and two had no difference. Extrathyroidal extension was found to be reduced in seven studies out of the 14 that examined it. Six other studies saw no effect. One study found increased extrathyroidal extension incidence overall, and another found it to be the case in patients younger than 45 years of age. Lymph node metastases were found to be reduced in several studies, while others found no difference. One study found increased central lymph node metastases in HT patients. As for prognoses, most studies found positive aspects. One study found an increased recurrence rate in HT patients, however, it did not have a relationship with deaths.

In conclusion, when managing HT or HT and PTC patients, HT patients should be monitored closely for suspicious nodules due to their frequent co-occurrence. Although the effect of HT on PTC has been shown to be mostly protective, multifocality is more common in those patients and, therefore, a total thyroidectomy should be favored. The high false positive rates of lymph node metastases in diagnostic methods should be kept in mind when considering lymph node dissection. Additional diagnostic procedures such as frozen section histology should be considered for verification.

## Introduction and background

Papillary thyroid carcinoma (PTC) is the most widespread endocrine malignancy, and its incidence has been rapidly rising [1]. It accounts for roughly 80% of all thyroid cancer cases [1]. It is categorized as well-differentiated cancer with the most favorable prognosis compared to other thyroid malignancies [2]. The main risk factor is considered to be radiation exposure [1]; however, the exact pathogenesis has not yet been established [3]. That being said, certain genetic alterations were identified to take part in the pathogenetic process: BRAF and RAS point mutations, and RET/PTC oncogene rearrangements [4]. It has been suggested that they both provoke and advance PTC, and the BRAF point mutation is the most frequent in such patients [4].

Hashimoto’s thyroiditis (HT), also called chronic lymphocytic thyroiditis (CLT) [5], is a common autoimmune endocrine disease, responsible for most cases of hypothyroidism in areas with adequate consumption of iodine [6]. HT has also been steadily on the rise [5]. It is histologically characterized by diffuse lymphocytic infiltration of the thyroid gland [7]. It is most commonly primary, wherein the cause is unknown, or secondary, due to iatrogenic causes such as immunomodulatory drugs [7]. The diagnosis was rare up to the late 1950s, and now its incidence ranks it among the most common autoimmune diseases [7].

Many studies describe associations between the two conditions - papillary thyroid carcinoma and Hashimoto thyroiditis [8,9]. The first study was published by Dailey et al. in 1955 [10]. Moreover, a multifold increase of PTC in HT patients has been reported [11-13]. Those two conditions share common factors such as more frequent occurrence in females, a history of exposure to ionizing radiation, and dietary iodine intake [14]. It is acknowledged that a connection between inflammation and cancer exists, and it has been presented as a possible but debatable explanation of the association between HT and PTC [14,15]. Additionally, both HT and PTC show some common dysregulated non-immune-linked genes involved in reactive oxygen species, oxidative stress, cell cycle, apoptosis, and DNA damage and repair [16]. 

Despite the observed associations, there is no consensus on whether and how HT causes PTC. There is also no definite evidence that HT coexistence affects PTC progression and outcomes. However, since they do tend to occur together [8-13], it is important to investigate their effect on the parameters that affect outcomes (such as tumor dimensions, angioinvasion, capsular infiltration, extrathyroidal extension, multifocality, and lymph node metastases [17]), which is important in guiding diagnostic and treatment decisions, with the ultimate goal of remission. In the present systematic review, we aim to present an overview of the available literature describing the effect of HT on PTC parameters and outcomes over the past 10 years.

## Review

Methods

**Figure 1:**
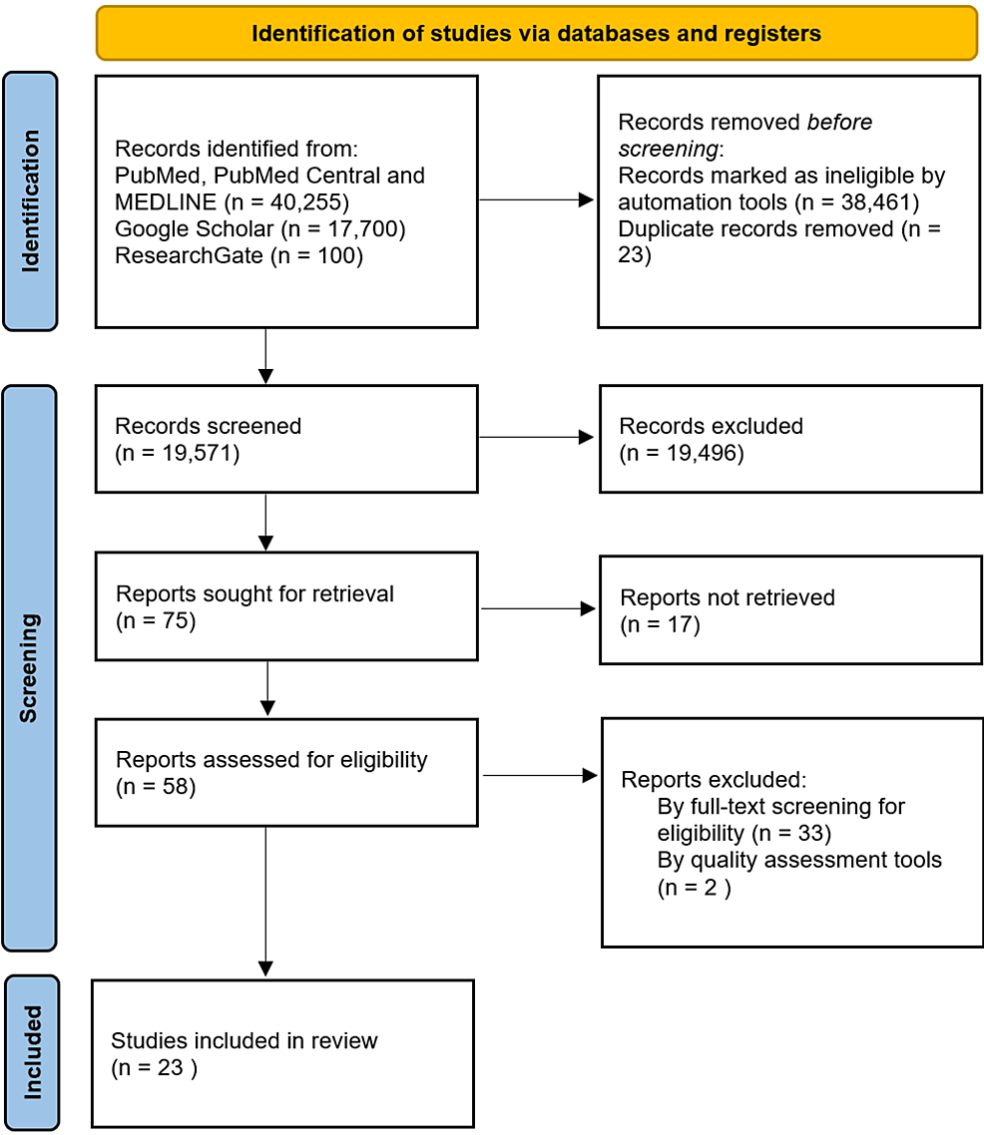
PRISMA flow diagram showing the article search and selection process. PRISMA: Preferred Reporting Items for Systematic reviews and Meta-Analysis

The authors followed the Preferred Reporting Items for Systematic reviews and Meta-Analysis (PRISMA) guidelines to conduct this systematic review [18]. The following databases were systematically searched by two reviewers: PubMed, ResearchGate, and Google Scholar. Medical subject heading (MeSH) terms were utilized for the search. The following keywords were utilized: “thyroid cancer,” “papillary carcinoma,” “papillary thyroid cancer,” “Hashimoto's thyroiditis,” “autoimmune thyroiditis,” “hypothyroidism,” “Hashimoto's disease,” “Hashimoto's syndrome,” “chronic lymphocytic thyroiditis,” “thyroiditis,” “staging,” “prognosis,” “prognostic factor,” “outcome,” and “recurrence.” AND and OR terms were utilized to create combinations with keywords to yield the most relevant results. Figure 1 below demonstrates the search methodology. The results were scanned from three databases manually by the first author and second author separately and then compared. Before applying filters, 58,055 total results were identified in the databases. The following figure depicts the PRISMA flow diagram (Figure 1).

Inclusion and Exclusion Criteria

Articles that describe the conditions surrounding the occurrence of PTC in CLT in human adults; articles must describe the effect on outcomes, recurrence, aggression, or prognosis in the clinical scenario; observational studies including case controls and case series were included. Only articles available in English and published in the last 10 years were selected. Articles published in journals that are not peer-reviewed, gray literature, animal studies, and in vitro studies were excluded.

Results

A total of 58,055 studies were found on PubMed, PubMed Central, MEDLINE, ResearchGate, and Google Scholar using the search terms. Automation tools such as filters were applied to match the inclusion and exclusion criteria. Afterward, the results were compared to remove duplicates. In total, 19,571 articles were subsequently screened. Fifty-eight out of the 75 chosen articles were retrieved, and finally, 24 studies were selected after full-text screening for eligibility. Those 24 studies were subject to quality assessment. The Newcastle-Ottawa tool was used to assess case-control and cohort studies. As a result, 23 studies passed the set percentage.

This systematic review includes data on 41,646 patients with PTC from 23 studies. Most of the factors examined below were either found to be affected positively by HT or not affected at all. The most positive observations were related to PTC tumor size, whereas multifocality was observed to be more so negatively affected by HT. Table 1 below summarizes selected studies.

**Table 1:** A summary of the studies examined in this review. HT: Hashimoto’s thyroiditis; LNM: lymph node metastases; PTC: papillary thyroid carcinoma; MACIS score: a prognostic score factoring in distant metastasis, patient age, completeness of resection, local Invasion, and tumor size; TNM: tumor, nodes, metastases (malignant tumor classification system); LN: lymph node

Study author and year	Study sample size	Country	Purpose of study	Results/conclusion
Molnár et al., 2019 [19]	262	Hungary	To evaluate clinicopathological and molecular connections of Hashimoto’s thyroiditis (HT) and Papillary thyroid cancer	Higher incidence of multifocality, lower incidence of LNM. It may be caused by extensive clinical follow-up due to enlarged lymph nodes in HT.
Jeong et al., 2012 [20]	1,357	Korea	To examine clinical features of concurrent HT and PTC.	PTC tumors in patients with HT are smaller and demonstrate extrathyroidal extension less frequently. Patients with PTC and HT are younger. HT was not determined to be a significant independent negative predictive factor for recurrence, despite it being associated with a reduced chance of recurrence.
Hanege et al., 2020 [21]	1,080	Turkey	To examine whether there is an association between HT and PTC and the effect of the coexistence of the two conditions on prognosis.	Increased risk of PTC multifocality in HT patients.
Ahn et al., 2011 [22]	675	Korea	To examine the clinical relationship between HT and PTC and its effect on clinical and pathological features.	Patients with both HT and PTC had better outcomes: lower MACIS score, smaller tumor size, less frequent lymph node metastases, and higher survival. However, the rates were not statistically significant.
Dobrinja et al., 2016 [23]	160	Italy	The effect of concurrent HT on PTC prevalence and prognosis.	HT is linked to a statistically significant lower PTC grade, lower chance of central LNM, angioinvasion, and capsular infiltration.
Konturek et al., 2013 [8]	7,545	Poland	To examine PTC and HT co-occurrence.	Metastases to lymph nodes in the central cervical compartment increased.
Liu et al., 2014 [24]	6,432	China	To examine the association between HT and PTC stages and lymph node metastases.	No association between HT and PTC lymph node metastases was found. HT may be linked to low-risk PTC.
Girardi et al., 2014 [25]	417	Brazil	To examine the relationship between HT and PTC and its effect on clinical and pathological features.	PTC tumors had lower staging, smaller size, and less common extraglandular involvement.
Babli et al., 2018 [26]	475	Canada	To examine the impact of HT on PTC.	Patients with coexistent PTC and HT had a higher share of TNM stage one, fewer patients with persistent disease. In patients older than 45 years of age, less persistent disease and less capsular invasion were observed. In patients younger than 45 years of age, more persistent disease and extrathyroidal extension occurred.
Liang et al., 2017 [27]	1,392	China	To examine the impact of HT on PTC clinicopathological features and prognosis.	Patients with coexistent HT and PTC had reduced tumor size, increased multifocality, reduced LNM, and better outcomes.
Zhu et al., 2016 [28]	763	China	To examine LNM risk in PTC and multifocal papillary thyroid carcinoma when coexistent with HT.	Reduced incidence of central LNM-HT determined to be an independent alleviating factor by multivariate analysis. However, multifocality and capsular invasion frequency was increased in HT.
Song et al., 2018 [29]	1,369	Korea	To evaluate the impact of LN dissection on outcomes for patients with coexistent PTC and HT. Additionally, to examine the effect of HT itself on outcomes.	Reduced metastatic LN ratio, structural recurrence, and persistence of PTC in HT patients. LN metastasis risk not reduced in HT patients.
Marotta et al., 2017 [30]	301	Italy	To examine the effect of HT on PTC outcomes.	HT was related to better PTC prognoses, reduced tumor size, reduced aggression, a higher chance of remission, and longer disease-free survival.
Jara et al., 2013 [31]	495	United States	To examine the effect of HT on PTC central LNM frequency.	Patients with both PTC and HT had a lower likelihood of central LNM, smaller tumors, lower staging, and lymphovascular invasion.
Zhang et al., 2014 [32]	8,524	China	To examine the impact of HT on PTC clinicopathological features and prognosis.	Patients with both PTC and HT had smaller tumor size, reduced extrathyroidal invasion, reduced lateral LNM, and lower staging
Yoon et al., 2012 [33]	195	Korea	To examine the impact of HT on PTC clinicopathological features.	Patients with both PTC and HT had smaller tumor size, reduced capsular invasion, and cental LNM.
Cordioli et al., 2013 [34]	94	Brazil	To examine the impact of HT on PTC clinicopathological features.	Patients with both PTC and HT had increased incidence of tumor multifocality, lower staging, and smaller tumor size.
Ryu and Yoon, 2020 [35]	850	Korea	To examine the effect of HT on PTC LNM.	HT was associated with tumor size less than 1 cm, low LNM, no extrathyroidal extension, and better disease-free survival.
Lee et al., 2020 [36]	2,928	Korea	To examine the impact of HT on PTC clinicopathological features and prognosis.	Patients with both PTC and HT had smaller tumors, increased extrathyroidal extension, and multifocality. Central LNM was decreased. Recurrence was lower.
Kwak et al., 2014 [37]	1,945	Korea	To examine the impact of HT on PTC clinicopathological and aggressive features.	HT patients had lower tumor staging and higher recurrence. No relationship with death.
Zhu et al., 2015 [38]	1,276	China	To examine the effect of HT on PTC central LNM and its possibility of predicting lateral LNM.	HT patients had comparatively lower central and lateral LNM. HT may be protective.
Carvalho et al., 2017 [39]	633	Brazil	To examine the effect of HT on PTC recurrence in patients with an excellent response to initial therapy.	The recurrence risk is not affected by HT in those patients.
Lun et al., 2013 [3]	2478	China	To examine the impact of HT on PTC clinical features and prognosis.	HT patients had smaller PTC tumors and lower staging.

Tumor Size

Twenty-two studies presented data on the effect of coexistent HT on PTC tumor size,where 12 studies demonstrated that HT is associated with a smaller tumor diameter (p < 0.05), while 10 studies suggest no statistically significant difference. No studies claim HT is associated with larger tumor size.

Angioinvasion

As for angioinvasion, 11 studies presented data, with only two of them suggesting a decreased incidence of angioinvasion, with the others finding no significant difference. No studies suggested increased incidence.

Capsular Infiltration

Five studies discussed capsular infiltration specifically, out of which, two claimed decreased occurrence, one suggesting increased occurrence, and one showing no significant difference. However, one of the studies distinguished between patients older than 45 years old and younger, with the former having decreased capsular infiltration and the latter having it increased.

Multifocality

Out of 19 studies that examined multifocality, 11 state that they found no statistically significant difference. However, seven studies suggest an increase in PTC multifocality incidence in HT patients. None of those studies found a decreased incidence of multifocality.

Extrathyroidal Extension

Fourteen studies investigated extrathyroidal extension. Seven of them found PTC extrathyroidal extension incidence significantly reduced in HT patients. Six studies found no correlation. One study presented increased incidence, and another showed the same but only in patients younger than 45 years of age.

Lymph Node Metastases

Twenty-two studies evaluated the association between HT and PTC lymph node metastases. Eleven studies have not found a statistically significant difference between HT and non-HT patients. Three studies observed decreased incidence of lymph node metastases in HT patients. Five studies demonstrate decreases in only central lymph node metastases. One study presents increased central lymph node metastases in HT patients. One study showed a decrease in lateral lymph node metastases while another suggests no significant difference in the same group of lymph nodes.

Overall Prognosis

Eleven studies commented on the overall prognosis including factors such as prognosis, recurrence, remission, disease-free survival, and disease persistence. Two studies suggested an overall better prognosis in HT patients with PTC compared to non-HT patients. One study found no negative association between CLT and PTC recurrence. One study observed decreased persistent disease. Two studies suggested increased disease-free survival. One study demonstrated decreased recurrence of disease. Three studies found no correlation between HT and PTC prognosis. Only one study observed increased recurrence in HT; however, it was not found to have a relationship with deaths.

Discussion

HT is an autoimmune tissue-specific disease that is histologically characterized by extensive lymphocytic infiltration, parenchymal atrophy, and fibrosis [27]. Long-term HT results in hypothyroidism, wherein thyroid-stimulating hormone (TSH) increases [27]. Cytologically, HT may even be misdiagnosed as PTC due to some similar nuclear features, including nuclear grooves and intranuclear inclusions [23,36].

PTC’s 10-year survival rates have been shown to be 93% and higher [20]. That being said, the overall excellent prognosis is affected by several factors, including tumor size, staging, metastases, extrathyroidal extension, age at diagnosis, timeliness of diagnosis, the extent of surgery, and radioiodine ablation [20]. The incidence of PTC and HT together has been reported to increase over the last 20 years, and the studies examined in this review have shown the incidence of HT in their PTC patients to be ranging from 14.2% to 56.5% [32,35]. The most notable results of this review show that the majority of studies found a significant decrease in tumor size in HT patients. However, several studies also observed more incidences of multifocality in HT patients. Even so, PTC still maintains an excellent prognosis, and many studies suggest that it’s even better with HT comorbidity.

It is not clear whether either one of the conditions causes the other, they both occur as a result of another trigger or they simply happen together by chance since they are both highly prevalent. However, their rather frequent coexistence has drawn attention and several hypotheses exist to attempt explaining the link.

Pathophysiological link

The pathophysiological link between HT and PTC has been explored in several studies. The BRAFV600E mutation is the most common in PTC [37]. In contrast, it was found that when HT was present with PTC, the occurrence of this mutation was lower [19,36,37]. Since the BRAFV600E mutation is associated with worse outcomes, its reduced occurrence in HT patients may account for a protective effect [37]. However, Molnár et al. observed that due to the scarce frequency of BRAF mutations and the increased multifocality they observed in HT patients, BRAF may not play a carcinogenic role in those patients’ tumor formation [19]. 

RET/PTC oncogenes are of note in HT as they were found in the majority of those patients, and they have been proposed to herald the presence of PTC [8]. Dobrinja et al. put forward that those oncogenes may denote HT as a precancerous condition [23]. The autoimmune process in HT may result in tumor destruction as well, leading to less aggressive tumor behavior and improved survival [20,23,33]. The presence of Fas and Fas ligand in HT follicular cells, which trigger the Fas-mediated apoptotic pathway, can destroy tumor cells [3,36].

Cytotoxic T cells, lymphokine-associated killer cells, and natural killer cells in HT may also participate in tumor cell destruction [3]. Cytotoxic T cells are able to secrete interleukin-1, which reduces tumor expansion and metastases [32]. Humoral immune mechanisms were also found to be engaged in HT patients with PTC [20]. Ahn et al. point out that chronic inflammation is generally known to play an important part in cancer development [22]. However, Lun et al. call attention to the lower prevalence of PTC in Graves disease, another thyroid autoimmune disorder, hence autoimmunity may not be the cause of PTC [3].

**Figure 2:**
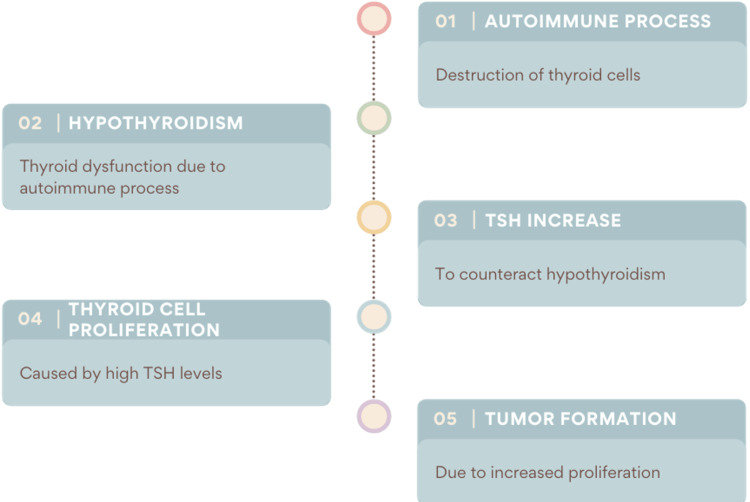
The potential effect of Hashimoto’s thyroiditis on papillary thyroid carcinoma. TSH: thyroid-stimulating hormone

It has been suggested that high TSH is linked to PTC since it promotes the proliferation of thyroid cells [3,27]. From this standpoint, Lun et al. concluded that the autoimmune process leads to hypothyroidism, which increases TSH, which in turn increases PTC risk [3]. Liang et al. also deduced that TSH may be the cause of PTC in HT patients [27]. However, it’s long-term HT that leads to a TSH increase, which could explain the resulting lack of difference between HT and non-HT PTC patients’ clinicopathological features in the prospective study by Carvalho et al. [39]. The amount of lymphocytic infiltrate may also differ in patients, which could potentially rationalize the disparity in some studies’ results [39]. Figure 2 depicts the effect of Hashimoto’s thyroiditis on papillary thyroid carcinoma.

Liu et al. point out that while chronic inflammation in the thyroid may trigger malignant changes, it may also create an environment that limits tumor expansion [25]. However, it would not be possible to test this hypothesis due to the absence of immunomodulating therapies that can be used to intervene [24]. Considering all these connections, Molnar et al. and Kwak et al. suggest that thyroiditis may be a precancerous condition [19,37]. Zhang et al. point out that increased iodine consumption has been linked to thyroid cancer and it may also affect the association between HT and PTC [32].

Protective effects of Hashimoto’s thyroiditis on papillary thyroid carcinoma

The results of the studies in this review show that, in general, patients who had both PTC and HT were predominantly female and younger than patients without HT.

Zhang et al. present the positive effects of HT on PTC to be smaller tumor size, decreased extrathyroidal extension, and decreased lateral lymph node metastases [32]. They suggested that this signals a better prognosis; however, the follow-up time in the study was not long enough to evaluate survival time [32].

Lun et al. and Liu et al. also found tumor size to be smaller in HT patients [3,24]. Lee et al.’s results were in agreement, showing smaller tumor size and decreased central lymph node metastases [36]. They note that HT patients undergo more central neck lymph node removals and more invasive diagnostic procedures [36]. Since imaging may show HT features as similar to PTC, they recommend performing a fine-needle aspiration biopsy to avoid unnecessary lymph node removal [36].

Kwak et al. found a decreased incidence of extrathyroidal extension [37]. Liang et al. found the protective effects of HT to be smaller tumor size and a lower rate of lymph node metastases [27]. Patients with HT and PTC were detected at the early stages of disease [27]. The autoimmune process in HT targets thyroid-specific antigens, limiting lymph node metastases [27]. The study by Song et al. confirmed a decrease in structurally persistent or recurrent disease [29].

Jeong et al. found a smaller tumor size and decreased extrathyroidal extension in HT patients [20]. However, HT was not found to be an independent negative predictive factor [20]. Jeong et al. acknowledge that this may be due to HT patients having other positive prognostic factors including female sex and younger age [20]. The study by Zhu et al. presented decreased incidence of extrathyroidal extension and lymph node metastases [38]. They also warn against unnecessary lymph node dissections [38]. Hanege et al. found an increased incidence of multifocality in HT patients and therefore suggest surgical intervention that considers this risk [21].

Ryu and Yoon found HT patients to have smaller tumors and decreased risk of extrathyroidal extension and lymph node metastases [35]. This supports the concept of HT, resulting in better prognoses [35]. Zhu et al. found a decreased incidence of central lymph node metastases in HT patients [28]. This distinction was more evident in multifocal tumors [28].

Jara et al., just as most of the other authors in this review, found tumor size to be smaller in HT patients [31]. Additionally, they observed a decreased incidence of angioinvasion, extrathyroidal extension, and central lymph node metastases [31]. Additionally, preoperative thyroxine usage diminished the incidence of central lymph node metastases even more [31].

Babli et al. drew a distinction between patients younger than 45 years and older than 45 years [26]. In HT patients older than 45 years of age, PTC capsular infiltration was decreased [26]. Both groups had decreased persistent disease incidence compared to the non-HT patients [26]. Girardi et al. found smaller tumor size in HT patients and decreased extrathyroidal extension [25]. They recognize that HT may be linked with better outcomes in some studies due to being associated with other factors that lead to an improved prognosis, rather than being a positive predictive factor by itself [25].

Marotta et al. observed increased remission rates and disease-free survival likelihood in HT patients [30]. Molnár et al. found that PTC lymph node metastases were significantly less common in HT patients [19]. They state that the effect of HT on lymph nodes, due to continuous immune activation and lymphatic hyperplasia, is lymphadenomegaly [19]. Therefore, they are attentively monitored [19]. This leads to more timely detection and treatment of PTC [19].

The study by Yoon et al. has demonstrated that smaller tumor size, decreased capsular infiltration, and decreased central lymph node metastases were seen in HT patients [33]. Based on those results, along with female preponderance and younger age, those authors expect a favorable prognosis [33].

Dobrinja et al. found a lower chance of central lymph node metastases, angioinvasion, and capsular infiltration, also mentioning that the autoimmune response may limit tumor growth [23]. PTC grade was also lower [23]. Cordioli et al. observed a smaller tumor size in HT patients [34]. Overall, there was an abundance of reported protective effects.

Detrimental effects of Hashimoto’s thyroiditis on papillary thyroid carcinoma

Konturek et al., in their study of 7,545 patients, found that HT was linked to a four-fold increase in central lymph node metastases [8]. Lee et al. found an increased incidence of both multifocality and extrathyroidal extension in HT patients [36]. Despite multifocality being considered related to central lymph node metastases, which affect prognosis, the prognosis of HT patients was not affected by multifocality in the aforementioned study [36].

Zhu et al. observed that HT resulted in increased capsular infiltration and multifocality [28]. Furthermore, multifocality was found to be twice as likely in HT patients with PTC [28]. Babli et al. found some negative effects of HT on PTC in the age group under 45 years of age; they had increased capsular infiltration, multifocality, and extrathyroidal extension [26]. That being said, despite the increased rates of those parameters, the younger patients’ outcomes overall were still better than the older group [26].

Molnár et al. observed that HT patients presented with more incidences of PTC multifocality [18]. The results of Liang et al. and Cordioli et al. were in agreement regarding increased multifocality [27,34]. Since multiple studies indicated a higher occurrence of multifocality, a total thyroidectomy should be the treatment of choice in patients with PTC in HT.

Additional outcomes

Morphologically, some studies in the present review recorded earlier pathological staging [3,34]. Several studies made the cytological distinction between the classic features of HT (widespread lymphocytic infiltration, parenchymal atrophy, and fibrosis) and the local lymphocytic infiltration inside or around a tumor, also known as tumor-associated lymphocytes [20,22,33,34]. This can be considered as a cause of the differing results in outcome factors.

Notably, ultrasonography had a higher false-positive rate in HT patients, showing more suspicious lymph nodes that were, in fact, simply affected by the autoimmune process [31]. Considering that, and the similarities of HT and PTC on fine-needle aspiration biopsy, it’s important to improve the diagnostic guidelines for HT patients. Otherwise, they may become subject to unnecessarily extensive lymph node dissection. However, there are proponents of prophylactic central lymph node dissection in any case of PTC and HT [8,27]. In contradiction, Jara et al. raise concern over complications since their rate is low only when the surgery is performed by high-volume, experienced surgeons [31]. Dobrinja et al. propose carrying out central lymph node dissection only based on intraoperative frozen section histology [23]. As for thyroid nodules in general, Zhang et al. recommend utilizing levothyroxine suppressive therapy and regular monitoring in young patients with HT and nodules less than one centimeter in size [32].

Several studies attempted to evaluate the difference in clinicopathologic features of PTC depending on the severity of HT [20,31]. They hypothesized that if HT improves prognosis, then higher severity should result in even better outcomes [20,31]. Especially since thyroxine would be more likely administered, reducing TSH and the autoimmune process [31]. However, no significant difference was found. HT patients may be subject to regular thyroid monitoring, which could explain the earlier detection and better prognoses.

Dobrinja et al. did not find a lower recurrence rate, longer disease-free survival, or a lower rate of distant metastases in HT patients, despite having a less aggressive disease course [23]. Similarly, Liang et al. did not find a statistically significant difference in overall survival in HT patients [27]. They explain that the overall excellent prognosis of PTC presents a challenge in investigating survival differences within subgroups [27]. Even so, after analyzing risk groups using the AMES stage and the prognostic score using the MACIS scoring system, it was found that HT patients had proportionately lower risk and better prognoses [27].

Limitations

There are several limitations to this systematic review. Only one study was prospective, the rest were retrospective and had limitations inherent to retrospective studies, wherein access to complete and accurate data may be limited. Patient selection was not identical across studies. Most studies were based on patients who had thyroidectomies, which accounts for a selection bias. HT itself is not an indication for surgery, so it was not possible to account for those patients. Additionally, it's not possible to accurately know how long the patient has had HT. Lymph node dissection was not performed in all patients, which may affect results. The role of iodine, which may be a confounding factor, was not examined in any studies. An ideal study would be prospective and examine all patients with nodular disease or the general population, with a long-term follow-up. However, it would be irrational to perform invasive procedures or operations on low-risk patients. Some papers included were published before 2018 and others were published after, which is significant as the TNM staging was updated in 2018 from the seventh to the eighth edition. This may result in the earlier papers reporting higher staging for the same degree of extracapsular extension.

## Conclusions

The aim of this review was to examine the effect of HT on PTC characteristics such as tumor dimensions, angioinvasion, capsular infiltration, multifocality, extrathyroidal extension, lymph node metastases, and outcomes. It was established that there is a high proportion of PTC patients who have HT. Many results showed a protective effect of HT by being associated with a smaller tumor size, reduced incidence of extrathyroidal extension, and decreased incidence of lymph node metastases. Some studies observed no impact on PTC characteristics. A selected few studies observed some detrimental effects of HT on PTC characteristics, most notably multifocality. It was also found that HT patients are more likely to have false-positive ultrasonography results regarding lymph node metastases. It’s important to keep all those factors in mind to guide the management of HT patients. The higher incidence of multifocality would make a total thyroidectomy more appropriate. It’s recommended to bear in mind the higher number of suspicious lymph nodes due to the autoimmune process and give them due attention to avoid unnecessary interventions. In patients who only have HT, monitoring with the possibility of PTC in mind is appropriate. To examine the coexistence of HT and PTC more definitively, extensive long-term prospective studies of the population are required.
